# Rapid functional divergence after small-scale gene duplication in grasses

**DOI:** 10.1186/s12862-019-1415-2

**Published:** 2019-05-02

**Authors:** Xueyuan Jiang, Raquel Assis

**Affiliations:** 1 Huck Institutes of the Life Sciences, Pennsylvania State University, University Park, PA USA; 2 Department of Biology, Pennsylvania State University, University Park, PA USA

**Keywords:** Gene duplication, Expression divergence, Neofunctionalization

## Abstract

**Background:**

Gene duplication has played an important role in the evolution and domestication of flowering plants. Yet little is known about how plant duplicate genes evolve and are retained over long timescales, particularly those arising from small-scale duplication (SSD) rather than whole-genome duplication (WGD) events.

**Results:**

We address this question in the Poaceae (grass) family by analyzing gene expression data from nine tissues of *Brachypodium distachyon*, *Oryza sativa japonica* (rice), and *Sorghum bicolor* (sorghum). Consistent with theoretical predictions, expression profiles of most grass genes are conserved after SSD, suggesting that functional conservation is the primary outcome of SSD in grasses. However, we also uncover support for widespread functional divergence, much of which occurs asymmetrically via the process of neofunctionalization. Moreover, neofunctionalization preferentially targets younger (child) duplicate gene copies, is associated with RNA-mediated duplication, and occurs quickly after duplication. Further analysis reveals that functional divergence of SSD-derived genes is positively correlated with both sequence divergence and tissue specificity in all three grass species, and particularly with anther expression in *B. distachyon*.

**Conclusions:**

Our results suggest that SSD-derived grass genes often undergo rapid functional divergence that may be driven by natural selection on male-specific phenotypes. These observations are consistent with those in several animal species, suggesting that duplicate genes take similar evolutionary trajectories in plants and animals.

**Electronic supplementary material:**

The online version of this article (10.1186/s12862-019-1415-2) contains supplementary material, which is available to authorized users.

## Background

Angiosperms, or flowering plants, compose one of the most evolutionarily and phenotypically diverse group of eukaryotes. Findings stemming from comparative genomic and experimental studies have led researchers to hypothesize that this extraordinary diversity is primarily a product of gene duplication events [1–3]. For one, duplicate genes are more abundant in angiosperms than in any other sequenced taxonomic group [2, 3], and differences in numbers of duplicates often contribute to genome sizes that differ by many orders of magnitude, even between closely related species [4, 5]. Second, a number of studies have shown that gene duplication can promote the origin of novel plant phenotypes [1, 3], and that it was likely a key driving factor in the domestication of flowering plants [6–9]. However, many of these findings are associated with studies of duplicates derived from whole-genome duplication (WGD) events, which occurred several times during the past 200 million years of angiosperm evolution [1, 10–15]. Yet substantial evidence shows that, in both plants and animals, duplicates deriving from WGD and small-scale duplication (SSD) events differ in quantifiable ways, such as evolutionary rate, essentiality, and function [11, 16–18]. Therefore, an open question is how SSD-derived genes in angiosperms evolve and are retained over long evolutionary timescales.

In the simplest case, SSD creates two copies of an ancestral single-copy gene. Considering directionality of duplication, the copy representing the ancestral gene is often called the “parent”, whereas the copy generated by duplication is termed the “child” [19, 20]. Four mechanisms may underlie the evolution and long-term retention of gene copies in such a scenario. First, under conservation, the ancestral function is preserved in each copy after duplication. Conservation may be due to negative selection acting to maintain a beneficial effect of increased gene dosage [21], or may simply arise as a consequence of nonallelic gene conversion between copies [2]. Second, under neofunctionalization, one copy preserves the ancestral function, whereas the other copy acquires a new function [21]. Neofunctionalization is hypothesized to occur as a result of positive selection acting on beneficial mutations that arise in one copy [21]. Third, under subfunctionalization, the ancestral function is divided between copies [22, 23]. Subfunctionalization is hypothesized to occur under either positive selection acting on mutations that optimize different subfunctions of each copy [24] or, more popularly, under neutrality if degenerative mutations impair different subfunctions of each copy [22]. Last, under specialization, rapid subfunctionalization is followed by neofunctionalization, resulting in both copies having distinct functions from one another and from their ancestral gene [25, 26].

Though examples of all of these hypothesized retention mechanisms exist in angiosperms [27–33], their relative abundances on a genome-wide scale remain unknown. One of the reasons for this gap in knowledge is the lack of methods for assessing functional divergence after gene duplication. To overcome this obstacle and distinguish among retention mechanisms of duplicate genes, researchers developed a phylogenetic approach that compares expression profiles between the ancestral single-copy gene in one species and the parent and child copies arising from a SSD event in a closely related sister species [20]. Application of their approach to RNA-seq data from two *Drosophila* species suggested that approximately 65% of duplicate genes underwent neofunctionalization [20]. Further analyses revealed that neofunctionalization often occurs within a few million years of duplication, results in acquisition of new functions by child copies that arose via RNA-mediated mechanisms, and generates testis-specific gene functions [20, 34]. In contrast, examination of RNA-seq data from eight mammals showed that only 33% of duplicate genes were retained by neofunctionalization [35]. The majority of duplicates were instead retained by conservation, and expression divergence was found to occur more gradually in mammals than in *Drosophila*, result in acquisition of new functions equally by parents and children, and generate a diversity of tissue-specific gene functions [35].

Natural selection may act more efficiently in *Drosophila* duplicate genes due to their much larger effective population sizes (*N*_e_; ~ 10^5^–10^6^) than mammals (~ 10^4^–10^5^; [36]), which may have contributed to the higher rates of expression divergence observed in *Drosophila* duplicate genes [20, 35]. In particular, the efficiency of selection is proportional to *N*_e_ × *s*, where *s* is the selective advantage of a beneficial mutation [37, 38]. Therefore, because angiosperms and mammals have comparable *N*_e_ (~ 10^4^–10^5^ for both taxa; [36, 39–41]), we might expect similar levels of expression conservation between duplicate genes of flowering plants and those of mammals.

In this study, we assess the genome-wide roles of duplicate gene retention mechanisms after SSD in three closely related self-pollinating [42–44] angiosperms in the Poaceae (grass) family: *Brachypodium distachyon*, *Oryza sativa japonica* (rice), and *Sorghum bicolor* (sorghum). *B. distachyon* and *O. sativa japonica* share a more recent common ancestor 40–54 million years ago (MYA), and the most recent common ancestor of all three species occurred 45–60 MYA [45–48]. Grasses represent an interesting evolutionary system because they are agriculturally important [49] and, thus, have undergone domestication events in their recent evolutionary histories. Further, these three grass species are ideal for comparison due to the availability of RNA-seq data from the same nine tissues (leaf, anther, endosperm, early inflorescence, emerging inflorescence, pistil, embryo, seed five days after pollination, and seed ten days after pollination) that were obtained in a single lab under similar experimental conditions [49]. Hence, we have a powerful toolkit with which to assess expression divergence after SSD in grasses.

## Results

### Retention mechanisms of SSD-derived duplicates in grasses

**Fig. 1:**
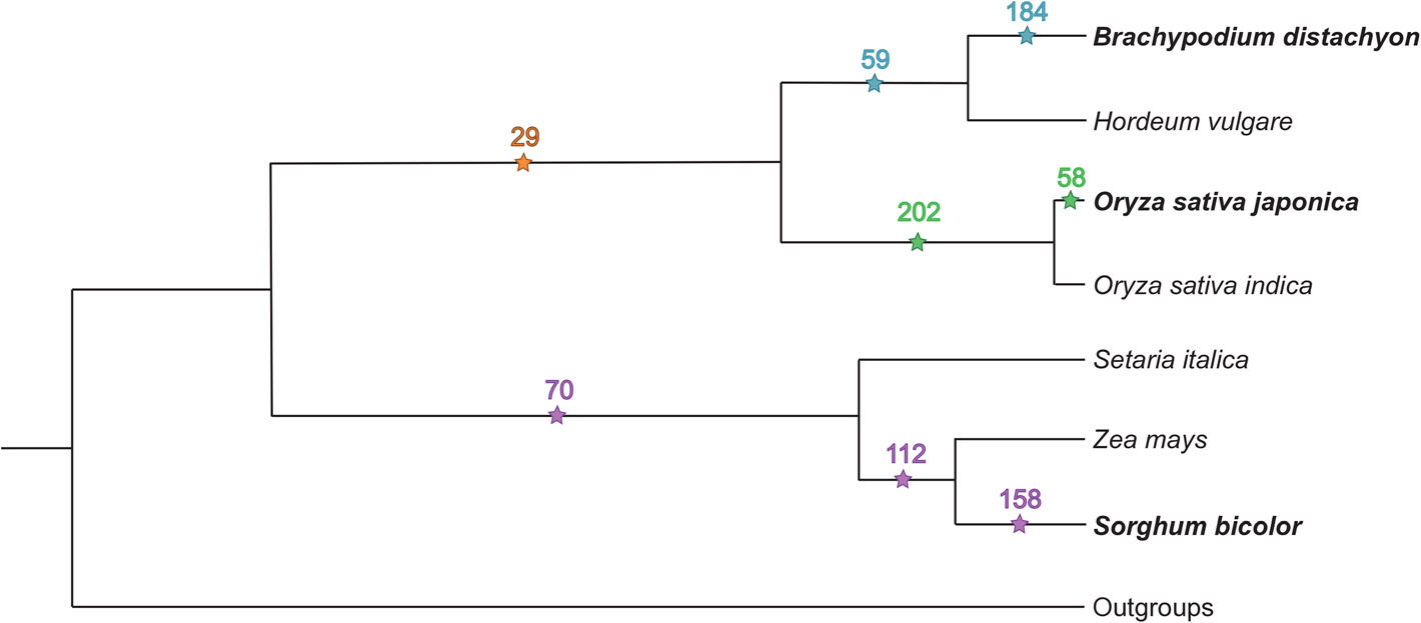
SSD-derived grass duplicate genes ascertained for our analysis. Numbers of SSD-derived duplicate gene pairs that arose along the *B. distachyon* (blue and orange stars), *O. sativa japonica* (green and orange stars), and *S. bicolor* (purple stars) lineages at specified divergence times on the monocot phylogeny. Outgroups used to polarize duplication events were *Musa acuminata, Arabidopsis thaliana*, *Carica papaya*, *Populus trichocarpa*, *Vitis vinifera*, *Solanum lycopersicum*, *Physcomitrella patens*, *Ostreococcus lucimarinus*, and *Chlamydomonas reinhardtii* (see Additional file 2: Figure S1 for full phylogeny)

A primary goal of our study was to understand how a pair of SSD-derived grass duplicate genes evolves and is retained after its emergence from a single-copy ancestral gene. Therefore, considering the phylogenetic tree depicted in Fig. 1, we were interested in pairs of duplicates that arose via SSD along the lineages of *B. distachyon* and *O. sativa japonica* after their divergence from *S. bicolor* (orange stars), *B. distachyon* after its divergence from *O. sativa japonica* (blue stars), *O. sativa japonica* after its divergence from *B. distachyon* (green stars), and *S. bicolor* after its divergence from *B. distachyon* and *O. sativa japonica* (purple stars). To identify such duplicates, we obtained a table of gene family sizes for 16 monocots and their full species phylogeny from the PLAZA 3.0 database [50]. Then, we used a maximum likelihood-based approach [51] to ascertain all duplications and losses that occurred along the monocot phylogeny. We applied parsimony rules to identify pairs of duplicates that arose along the branches indicated in Fig. 1 (see Additional file 2: Figure S1 for full tree). It is important to note that the most recent WGD event in monocots occurred approximately 65 MYA [14], which is before the divergence of *B. distachyon, O. sativa japonica*, and *Sorghum bicolor*. Therefore, given the size of the monocot tree and number of outgroups considered, the duplications that we extracted with this approach are more likely to be created by SSD rather than WGD events. Next, we required that both duplicate genes, as well as their single-copy ancestral gene in the closer of the two sister species considered, be expressed in at least one tissue (see Methods for details). This analysis yielded 272 SSD-derived gene pairs in *B. distachyon* (Additional file 1: Table S1), 289 pairs in *O. sativa japonica* (Additional file 1: Table S2), and 340 pairs in *S. bicolor* (Additional file 1: Table S3; Fig. 1). Using sequence and synteny information, we inferred the most likely parent and child copy for each pair of duplicates in this dataset (see Methods for details).

To classify retention mechanisms of SSD-derived grass duplicate genes, we applied the phylogenetic method developed by Assis and Bachtrog [20] to expression profiles constructed from RNA-seq data in nine tissues [49] of single-copy, ancestral, parent, and child genes of *B. distachyon*, *O. sativa japonica*, and *S. bicolor*. In particular, this method [20] first utilizes the distribution of Euclidian distances between expression profiles of single-copy genes to establish a cutoff that represents the expected expression divergence between two species. Next, it computes Euclidian distances between ancestral and parent expression profiles, ancestral and child expression profiles, and ancestral and combined parent-child expression profiles. Last, it classifies retention mechanisms of each pair of duplicates based on phylogenetic rules. Briefly, the expression profile of the ancestral gene is expected to be similar to those of both the parent and child under conservation, to those of one copy but not the other under neofunctionalization, and to those of neither copy under subfunctionalization or specialization. Distinguishing between subfunctionalization and specialization requires an additional comparison of ancestral and combined parent-child expression profiles. Similarity between these expression profiles suggests that the function of the ancestral gene was subdivided between parent and child copies due to subfunctionalization, whereas dissimilarity points to functional divergence among all three genes due to specialization [20].

Application of the described classification approach [20] uncovered similar proportions of each retention mechanism among *B. distachyon*, *O. sativa japonica*, and *S. bicolor* SSD-derived duplicates (Table 1 and Additional file 1: Tables S1–3). Therefore, it appears that genes in all three grass species traverse similar evolutionary paths after SSD. In total, 60.6% of SSD-derived grass duplicates are conserved, 23.8% are neofunctionalized, 0.4% are subfunctionalized, and 15.2% are specialized. Hence, conservation is the most prevalent retention mechanism, indicating that SSD typically results in increased gene dosage in grasses. This level of functional conservation is higher than observed in *Drosophila* [20] and similar to that observed in mammals [35]. Thus, our observation is consistent with the smaller *N*_e_ of grass and mammalian species compared with *Drosophila* [36, 39–41].

**Table 1:** Classified retention mechanisms of SSD-derived grass duplicate genes

	*B. distachyon*	*O. sativa* japonica	*S. bicolor*
Conservation	170	158	218
Neofunctionalization (parent, child)	60 (17, 43)	80(24, 56)	74(8, 66)
Subfunctionalization	1	1	2
Specialization	41	50	46

### Contribution of duplication mechanism to expression divergence of SSD-derived grass duplicates

Despite a prominent role of conservation, over one-third of SSD-derived grass duplicate genes undergo expression divergence, most of which occurs asymmetrically via neofunctionalization. This pattern of asymmetric expression divergence is consistent with findings in both *Drosophila* [20] and mammals [35]. However, as in *Drosophila* [20] but not mammals [35], neofunctionalization in grasses is also biased in that approximately 72% of *B. distachyon*, 70% of *O. sativa japonica*, and 89% of *S. bicolor* neofunctionalized genes are child copies (Table 1 and Additional file 1: Tables S1-S3). In *Drosophila*, this bias was associated with RNA-mediated duplication [20, 34], which produces child copies lacking the introns and regulatory elements of their ancestral genes. The new genomic context of RNA-mediated child duplicates may increase their likelihood of possessing or acquiring novel gene functions [52]. Therefore, we hypothesized that RNA-mediated duplication may contribute to biased neofunctionalization of children in grasses as well. To test this hypothesis, we compared observed and expected counts of DNA- and RNA-mediated duplicates retained by conservation, neofunctionalization of parents, neofunctionalization of children, and specialization (Table 2; see Methods for details). Indeed, there is an overrepresentation of RNA-mediated duplicates retained by neofunctionalization of children (*P* = 0.01, χ^2^ test; see Methods for details), but not by any other mechanism. This finding indicates that RNA-mediated duplication is more likely to generate children with novel functions in grasses. Moreover, because this pattern exists in both grasses and *Drosophila* [20], it is possible that RNA-mediated duplication acts as a reservoir of functional innovation across many diverse taxonomic groups.

**Table 2:** Observed (expected) DNA- and RNA-mediated SSD-derived duplicates by retention mechanism

	DNA-mediated	RNA-mediated	*P*
Conservation	464 (455.98)	28 (36.02)	0.17
Neofunctionalization of parent	39 (38.00)	2 (3.00)	0.55
Neofunctionalization of child	126 (134.39)	19 (10.61)	0.01
Specialization	80 (80.63)	7 (6.37)	0.79

If RNA-mediated duplication contributes to neofunctionalization in grasses, then we might expect expression divergence to occur either as a byproduct of SSD or soon afterward. Therefore, next we were interested in ascertaining the timing of expression divergence after SSD in grasses. If expression divergence is rapid, then we expect the frequencies of retention mechanisms to be similar among duplicates that arose at different time points in monocot evolution, as was observed in *Drosophila* [20]. Alternatively, if expression divergence occurs more gradually after SSD, then we expect higher frequencies of conservation in duplicates that arose more recently and higher frequencies of divergence in those that arose more distantly in the past, as was observed in mammals [35]. To address this question in grasses, we divided the duplicates in our dataset into three age classes based on when SSD occurred along the monocot phylogeny and compared observed and expected counts of retention mechanisms in each age class (Additional file 2: Tables S4–6; see Methods for details). Consistent with findings in *Drosophila* [20], but not in mammals [35], proportions of retention mechanisms are similar among duplicates that arose by SSD at different time points in all three species. Therefore, it appears that functional divergence of SSD-derived grass duplicates often occurs either as a consequence of duplication or shortly afterward.

### Sequence- and tissue-specific correlates with expression divergence of SSD-derived grass duplicates

**Fig. 2:**
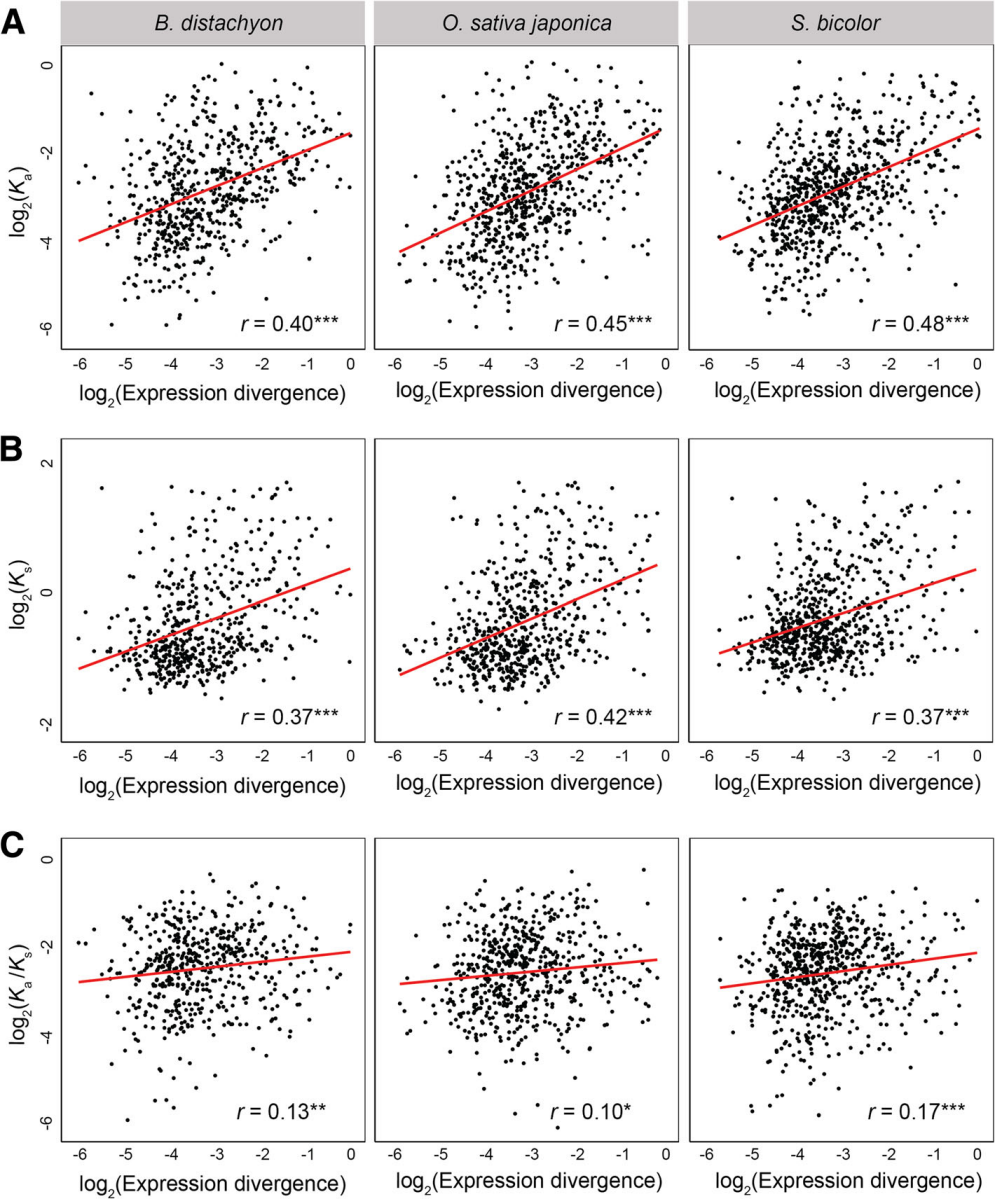
Relationship between expression and protein-coding sequence divergence rates of SSD-derived grass duplicate genes. Scatterplots showing correlations between expression divergence (Euclidian distance) and (**a**) nonsynonymous sequence divergence (*K*_a_), (**b**) synonymous sequence divergence (*K*_s_), and (**c**) nonsynonymous/synonymous sequence divergence (*K*_a_/*K*_s_) rates of SSD-derived duplicate genes in *B. distachyon* (left), *O. sativa japonica* (middle), and *S. bicolor* (right). The best-fit linear regression line is shown in red, and Pearson’s correlation coefficient (r) is provided at the bottom right, for each panel. *P < 0.05, **P < 0.01, ***P < 0.001

Previous studies have demonstrated that expression divergence is often positively correlated with protein-coding sequence divergence of duplicate genes in many species [20, 53–58]. To assess this relationship in grasses, we calculated Pearson’s correlation coefficients (*r*) between expression divergence (Euclidian distance) and nonsynonymous sequence divergence (*K*_a_), synonymous sequence divergence (*K*_s_), and nonsynonymous-to-synonymous sequence divergence (*K*_a_/*K*_s_) rates of each SSD-derived duplicate gene and its ancestral gene in *B. distachyon*, *O. sativa japonica*, and *S. bicolor* species (Fig. 2; see Methods for details). In all three species, there are moderately strong positive correlations between expression divergence and *K*_a_ (Fig. 2a; *r* = 0.40 − 0.48; *P* < 0.001 for all comparisons, *t* tests; see Methods for details), and between expression divergence and *K*_s_ (Fig. 2b; *r* = 0.37 − 0.42; *P* < 0.001 for all comparisons, *t* tests; see Methods for details), as well as a weak positive correlation between expression divergence and *K*_a_/*K*_s_ (Fig. 2c; *r* = 0.10 − 0.17, *P* < 0.05 for all comparisons, *t* tests; see Methods for details). Thus, expression divergence of SSD-derived duplicates is significantly associated with protein-coding sequence divergence rates, suggesting that expression patterns and encoded proteins of grass duplicate genes evolve in tandem.

**Fig. 3:**
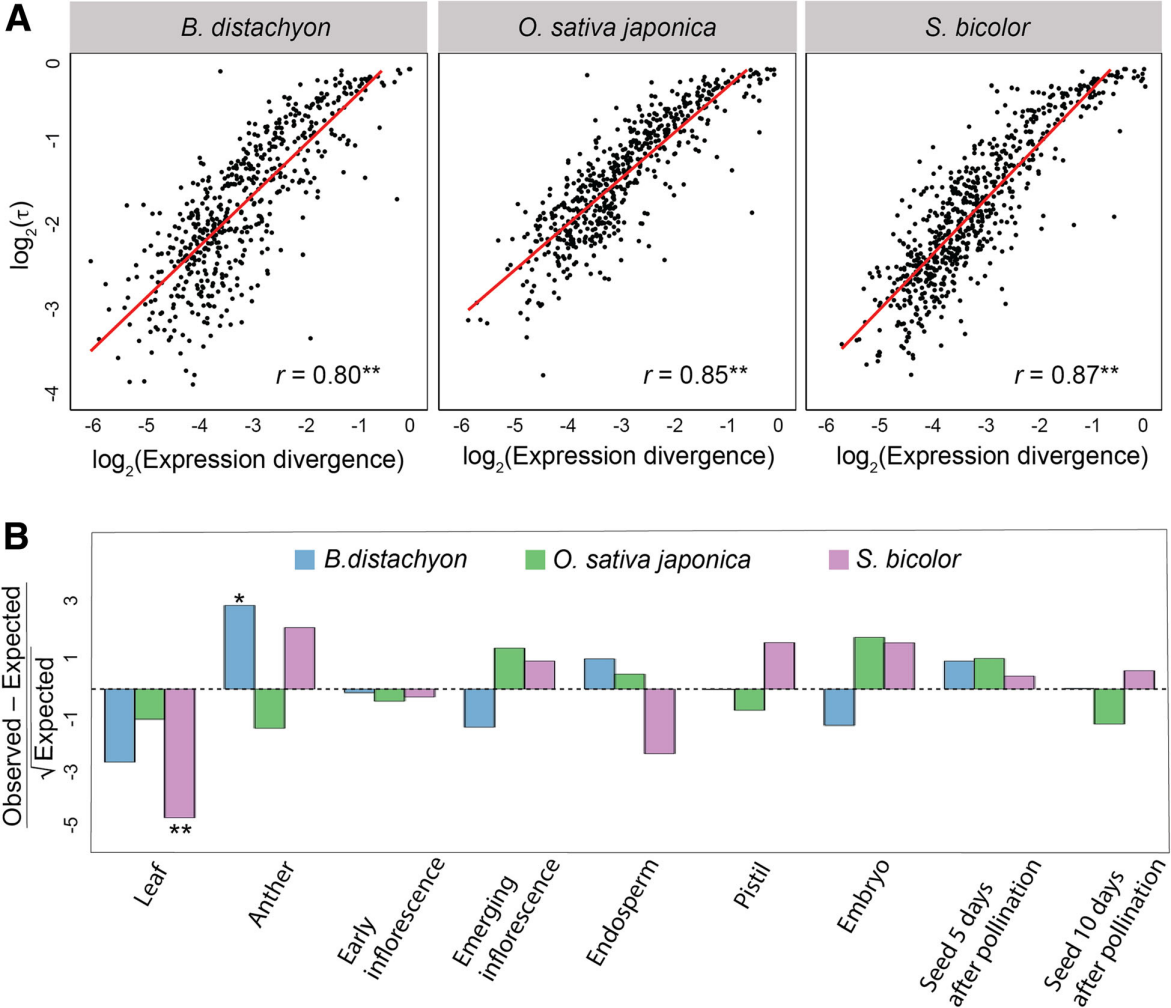
Relationship between expression divergence and tissue specificity of SSD-derived grass duplicate genes. **a** Scatterplot showing correlation between expression divergence (Euclidian distance) and tissue specificity (*τ*) of SSD-derived duplicate genes in *B. distachyon* (left), *O. sativa japonica* (middle), and *S. bicolor* (right). The best-fit linear regression line is shown in red, and Pearson’s correlation coefficient (*r*) is provided at the bottom right, for each panel. **b** Comparisons of observed counts of primary tissues of duplicate genes relative to those expected based on proportions of primary tissues of single-copy genes. Positive values represent overrepresentations, and negative values underrepresentations, relative to expectations. *P < 0.05, **P < 0.001

Moreover, expression divergence of SSD-derived duplicate genes is associated with increased tissue specificity in both *Drosophila* [20] and mammals [35]. To assess this relationship in SSD-derived grass duplicates, we computed Pearson’s correlation coefficients (*r*) between expression divergence (Euclidian distance) of each duplicate gene from its ancestral copy and its tissue specificity index *τ* ([59]; see Methods for details) in *B. distachyon*, *O. sativa japonica*, and *S. bicolor* (Fig. 3a). Consistent with results in *Drosophila* [20] and mammals [35], there is a strong positive correlation between tissue specificity and expression divergence of SSD-derived duplicate genes in all three grass species (*r* = 0.80 − 0.87; *P* < 0.001 for all comparisons, *t* tests; see Methods for details). Thus, increased expression divergence of SSD-derived grass duplicates is associated with greater tissue specificity.

Whereas SSD-derived duplicate genes in *Drosophila* are primarily testis-specific [20, 60–62], those in mammals are expressed specifically in a diversity of tissues [35]. Therefore, our next question was whether there are particular tissues in which SSD-derived duplicates tend to be expressed in grasses. To answer this question, we designated the tissue in which each gene has its highest expression as its primary tissue, and compared the observed primary tissues to those expected based on primary tissues of single-copy genes (Fig. 3b; see Methods for details). After correcting for multiple comparisons (see Methods for details), our analysis yielded two significant findings. First, there is an underrepresentation of leaf-expressed duplicates in *S. bicolor* (*P* = 1.84 × 10^−6^, binomial test; see Methods for details). Because leaf is the only tissue assayed that is not related to reproduction, this result suggests that duplicates in *S. bicolor* are typically expressed in reproductive tissues. Second, we discovered an overrepresentation of anther-expressed duplicates in *B. distachyon* (*P* = 0.02, binomial test; see Methods for details). Because the anther produces pollen grains [63], this result suggests that SSD-derived *B. distachyon* duplicates are involved in male-specific reproduction, as is common in many animal species [20, 35, 60–62, 64–66]. Therefore, SSD may be associated with reproduction in plants, as it is in animals.

## Discussion

Despite the abundance of duplicate genes in angiosperms, and their prominent roles in evolution [1, 3, 6–9], their paths from genetic redundancy to functional divergence and long-term retention remain unclear. Studies in several animal species have uncovered evidence of rapid and asymmetric sequence and expression divergence after duplication that is consistent with natural selection [20, 35, 57, 67–70]. However, many angiosperms are unique in that they are self-pollinating, which may reduce their adaptive potentials [71–74], and therefore hinder the evolutionary divergence of duplicate genes. Yet, largely due to the absence of approaches for assessing functional divergence after duplication until recently [20], no genome-wide studies have been performed to address how duplicate genes in angiosperms evolve and are retained over long evolutionary timescales. Further, previous studies in angiosperms have primarily focused on WGD-derived duplicates, whereas little emphasis has been placed on describing evolution after SSD. Therefore, our study represents the first genome-scale analysis of functional evolution after SSD in angiosperms.

Examination of expression profiles across nine tissues of *B. distachyon*, *O. sativa japonica*, and *S. bicolor* revealed that functional conservation is the primary long-term outcome of SSD in grasses. Conservation of duplicate genes may either be a product of negative selection that acts to preserve the ancestral function in both copies due to the benefits of increased gene dosage [2, 21], or a consequence of slowed functional divergence due to a decreased efficiency of selection [37, 38] if conservation is the result of nonallelic gene conversion. Either one or both of these mechanisms may hamper evolutionary divergence of duplicate genes in grasses. In particular, though our study focused on SSD, analyses of WGD often point to increased gene dosage as a mechanism for duplicate gene retention in plants [27]. On the other hand, levels of conservation in grasses are higher than those in *Drosophila* [20], and similar to those in mammals [35], consistent with predictions based on differences in *N*_e_ among these taxa [36]. Therefore, the comparison among levels of conservation in *Drosophila*, mammals, and grasses provides additional support for a role of natural selection in evolution after gene duplication across diverse taxonomic groups.

Though our analysis suggests that most grass duplicates are functionally conserved, they also indicate that a large proportion of SSD-derived duplicates may have experienced functional divergence. Previous studies in *Arabidopsis thaliana* demonstrated that SSD-derived duplicates have greater sequence and expression divergence rates than WGD duplicates of the same age [17, 75], which may be attributed to relaxed constraint [17]. Therefore, it is not surprising that SSD-derived duplicates in the species considered here may have diverged functionally from their ancestral state, and it is possible that an analogous study of WGD-derived duplicates would reveal a similar trend to that observed in *A. thaliana*. Moreover, we found that expression divergence of SSD-derived grass duplicates primarily occurs asymmetrically via neofunctionalization, as has been uncovered in both *Drosophila* [20] and mammals [35]. This finding is also consistent with the increased prevalence of neofunctionalization among *A. thaliana* duplicates generated by SSD [11]. Therefore, asymmetric evolutionary divergence appears to be a common outcome of SSD in both plant and animal species. However, neofunctionalization often occurs in child copies and is associated with RNA-mediated duplication in grasses, as in *Drosophila* [20], but not in mammals [35]. Further, evolutionary fates of grass duplicates are reached quickly after duplication, also consistent with findings in *Drosophila* [20], but not in mammals [35]. Together, these results support the hypothesis that neofunctionalization may often occur as a byproduct of SSD itself, perhaps due to the placement of RNA-mediated duplicates in novel genomic contexts without their ancestral regulatory elements [52]. Thus, aside from their slower divergence rates, the evolutionary trajectories of grass duplicates more closely mirror those of *Drosophila* [20] than mammals [35]. This is somewhat surprising because the *N*_e_ of grass species are smaller than those of *Drosophila* species [36, 39–41]. However, in mammals, functional divergence often occurs over longer evolutionary time [35], suggesting that neofunctionalization is only biased toward child copies when it happens rapidly. This is not unexpected, given that conserved duplicates are initially redundant and, thus, the probabilities of divergence of parent and child copies over time should be equal. Therefore, this comparison further highlights the role of asymmetric duplication events, such as those that are RNA-mediated, in asymmetric divergence and child-biased neofunctionalization.

Assessment of expression divergence of SSD-derived grass duplicate genes revealed that it is positively correlated with protein-coding sequence divergence and tissue specificity. Moreover, in *B. distachyon*, we found an enrichment of duplicates highly expressed in anther, which is the tissue that produces pollen in flowering plants. This finding is consistent with those in *A. thaliana* RNA-mediated duplicates [76, 77] and supports the “out of the pollen” hypothesis, in which new plant genes originate from the vegetative nucleus of the mature pollen due to increased activities of transposable elements [78]. Because anther is analogous to testis in animals, our result is also synonymous with the “out of the testis” hypothesis, which posits that new genes often emerge with testis-related functions and acquire novel functions over time [20, 79] and is supported by data in many species [20, 35, 60–62, 64–66]. Several hypotheses have been proposed to explain the male-biased origin of new genes, including increased mutation rates due to greater numbers of germline cell divisions in male tissues [80], positive selection due to sexual selection [81, 82], and relaxed negative selection due to reduced functional pleiotropy [82–84]. However, as in animals (e.g., [20, 35, 79]), any of these proposed mechanisms may contribute to the male-biased origin of duplicate genes in grasses. In particular, the increased mutation rate hypothesis [80] is consistent with more cell divisions during pollen than ovule production in grasses [85, 86], positive selection [81, 82] with the positive correlation between expression divergence and protein-coding sequence divergence of duplicates (Fig. 2), and negative selection [82–84] with the positive correlation between expression divergence and tissue specificity of duplicates (Fig. 3a). Therefore, comparison of our findings in grasses to those in diverse animal species [20, 35, 60–62, 64–66, 70] highlights a universal role for gene duplication in the origin of male-specific phenotypes across plant and animal kingdoms.

## Conclusions

Gene duplication is thought to be a key driver of phenotypic innovation. However, despite the abundance of duplicate genes in grasses, little is known about their evolution. In this study, we compare the gene expression profiles of SSD-derived genes in three closely related grass species to uncover their evolutionary trajectories after duplication. Our results suggest that, whereas most grass duplicates retain their ancestral gene functions, many rapidly acquire new functions. Moreover, consistent with findings in animals, many grass duplicate genes display male-biased expression patterns. Therefore, gene duplication may play a universal role in the origin and evolution of male-specific phenotypes.

## Methods

### Identification of single-copy and duplicate genes

Reference genome annotation and sequence data from *B. distachyon* (version 1.2; [87]), *O. sativa japonica* (version 1.0; [88]), and *S. bicolor* (version 1.4; [47]), as well as a table of protein-coding gene family sizes for 16 monocots, were downloaded from PLAZA 3.0 [50] at https://bioinformatics.psb.ugent.be/plaza/. Gene families consisting of one copy in *B. distachyon*, *O. sativa japonica*, and *S. bicolor* were considered as single-copy genes. In total, there are 5132 single-copy genes annotated in *B. distachyon*, 11,672 single-copy genes annotated in *O. sativa japonica*, and 6724 single-copy genes annotated in *S. bicolor*. Removal of lowly-expressed genes (see *Sequence and expression analyses*) yielded 4769 single-copy genes in *B. distachyon*, 5439 single-copy genes in *O. sativa japonica* and 5976 single-copy genes in *S. bicolor* that we used in tissue enrichment test. There are 3466 annotated 1:1 orthologs in *B. distachyon* and *O. sativa japonica,* 3166 annotated 1:1 orthologs in *B. distachyon* and *S. bicolor* and 3154 annotated 1:1 orthologs in *O. sativa japonica* and *S. bicolor*. Removal of lowly-expressed genes (see *Sequence and expression analyses*) yielded 3269 1:1 orthologs in *B. distachyon* and *O. sativa japonica*, 3024 1:1 orthologs in *B. distachyon* and *S. bicolor* and 3015 1:1 orthologs in *O. sativa japonica* and *S. bicolor*

To identify pairs of duplicate genes that arose via SSD along designated branches shown in Fig. 1 (full tree depicted in Additional file 2: Figure S1), we used the maximum-likelihood method Count [51] to estimate rates of duplications and losses along the monocot phylogeny downloaded from PLAZA 3.0 [50] and perform asymmetric Wagner parsimony using these rates [89]. In total, this approach yielded 391 pairs of duplicate genes that arose along the *B. distachyon* lineage, 478 pairs of duplicate genes that arose along the *O. sativa japonica* lineage*,* and 462 pairs of duplicate genes that arose along the *S. bicolor* lineage*.* After removing lowly-expressed genes (see *Sequence and expression analyses*), we obtained 272 pairs of *B. distachyon* duplicates, 289 pairs of *O. sativa japonica* duplicates, and 340 pairs of *S. bicolor* duplicates (see Fig. 1). To assess directionality of duplications and assign parent and child copies, we used tables of orthologs from OrthoMCL [90], TribeMCL [91], and i-ADHoRE [92] that were downloaded from the PLAZA 3.0 database [50]. When orthology predictions from all three methods were available yet conflicting, we applied a majority-voting scheme to infer the most likely orthologs. When predictions from only two methods were available and conflicting, we prioritized OrthoMCL orthologs above all others, and i-ADHoRE above TribeMCL.

### Sequence and expression analyses

We performed all sequence alignments between duplicates and ancestral single-copy genes using MACSE [93], which accounts for frameshifts and stop codons. We estimated *K*_a_, *K*_s_, and *K*_a_/*K*_s_ using the codeml package in PAML 4.0 [94] with runmode = − 2, model = 0, and NSsites = 0. To avoid saturation at synonymous sites, we only considered genes with *K*_s_ < 3. Tables containing expression abundances estimated in transcripts per million (TPM) from RNA-seq data of protein-coding genes in nine tissues (leaf, anther, endosperm, early inflorescence, emerging inflorescence, pistil, embryo, seed five days after pollination, and seed ten days after pollination; [49]) of *Brachypodium distachyon*, *Oryza sativa japonica*, and *Sorghum bicolor* were downloaded from Expression Atlas at https://www.ebi.ac.uk/gxa/home. These RNA-seq data were quantified with HTSeq 0.6 [95], which only counts reads that unambiguously map to a single gene, thereby minimizing the probability of incorrect mapping between duplicate gene copies. Data were then log-transformed, and genes with log_2_(TPM + 1) < 1 in all nine tissues were removed, as such genes are expressed at low levels that may be attributed to transcriptional noise. We estimated the expression breadth of each gene with the tissue specificity index *τ* [59], which is defined as $\tau =\frac{\sum_{i=1}^N\left(1-{x}_i\right)}{N-1}$, where *x*_*i*_ represents the expression level in the *i*^*th*^ tissue normalized by the maximal expression value. The range of *τ* is from 0 to 1, with larger *τ* signifying greater tissue specificity.

We classified retention mechanisms of duplicate genes in our dataset using the CDROM R package [96], which implements Assis and Bachtrog’s phylogenetic approach [20]. In particular, CDROM takes as input tables of expression measurements for multiple conditions in two sister species, lists of orthologous single-copy genes in the two sisters, and a list of parent and child duplicate gene pairs in one sister and their ancestral genes in the second sister. We used *B. distachyon* as the sister species to *O. sativa japonica* and *S. bicolor* and applied CDROM to the RNA-seq data described above, which consists of log-transformed TPMs for genes in nine tissues of *B. distachyon*, *O. sativa japonica*, and *S. bicolor* [49]. CDROM first calculates Euclidian distances between expression profiles of orthologous single-copy genes (*E*_S1, S2_), expression profiles of parent and child duplicate genes and the ancestral gene (*E*_P, A_ and *E*_C, A_), and combined expression profiles of both duplicate genes and the ancestral gene (*E*_P + C, A_). Next, it uses a user-specific cutoff for *E*_S1, S2_ (*E*_div_) to classify retention mechanisms of duplicates. Specifically, duplicates with *E*_P, A_ ≤ *E*_div_ and *E*_C, A_ ≤ *E*_div_ are classified as functionally conserved; those with either *E*_P, A_ ≤ *E*_div_ and *E*_C, A_ > *E*_div_ or *E*_C, A_ ≤ *E*_div_ and *E*_P, A_ > *E*_div_ as neofunctionalized; those with *E*_P, A_ > *E*_div_, *E*_C, A_ > *E*_div_, and *E*_P + C, A_ ≤ *E*_div_ as subfunctionalized, and those with *E*_P, A_ > *E*_div_, *E*_C, A_ > *E*_div_ and *E*_P + C, A_ > *E*_div_ as specialized. We used distributions of Euclidian distances between gene expression profiles to choose *E*_div_ for each species (Additional file 2: Figure S2).

### Determination of DNA- and RNA-mediated duplication mechanisms

Exon counts for parent and child duplicates were obtained from genome annotation files (*B. distachyon* version 1.2 [87], *O. sativa japonica* version 1.0 [88], and *S. bicolor* version 1.4 [47]) downloaded from the PLAZA 3.0 database [50]. The child was considered as arising through DNA-mediated duplication when the parent and child copies both have multiple exons, and through RNA-mediated duplication when the parent copy has multiple exons and the child copy has one exon. When both the parent and child have one exon, the mechanism was considered to be unknown (43 pairs in B. *distachyon*, 39 pairs in *O. sativa japonica*, and 50 pairs in S. *bicolor*). Genes with unknown duplication mechanisms genes were not used in the analysis presented in Table 2.

### Statistical analyses

We performed all statistical analyses in the R software environment [97]. χ^2^ tests were used to compare observed and expected DNA- and RNA-mediated duplicates retained through different mechanisms (Table 2), as well as observed and expected retention mechanisms of duplicates in different age groups (Additional file 2: Tables S4–6). Expected counts of DNA- and RNA-mediate duplicates were obtained by multiplying the number of duplicates retained by each mechanism by total proportions of DNA- and RNA-mediated duplicates, respectively. Expected counts of retention mechanisms of duplicates in different age groups were obtained by multiplying the number of duplicates retained by each mechanism by total proportions of duplicates in different age groups. Significance of Pearson’s correlation coefficients depicted in Fig. 2 were assessed via Student’s *t* tests. Two-tailed binomial tests were implemented to compare observed counts of highest-expressed duplicates relative to their expected probabilities. Each binomial test was performed by setting the number of trials as the total number of duplicates, the number of successes as the number of highest-expressed duplicates in the tissue of interest, and the probability of success as the frequency of single-copy genes in the tissue of interest. *P*-values from binomial tests were Bonferroni-adjusted to correct for the nine comparisons performed.

## Additional files

Additional file 1: **Table S1.** Classification of all duplicate gene pairs with Ediv values in *Brachypodium distachyon.* **Table S2.** Classification of all duplicate gene pairs with Ediv values in *Oryza sativa japonica.* **Table S3.** Classification of all duplicate gene pairs with Ediv values in *Sorghum bicolor*. **Tables S1–3.** Contain classifications of duplicate gene pairs in the three grass species. (XLSX 134 kb)

Additional file 2: **Table S4.** Observed (expected) counts of *B. distachyon* retention mechanisms by SSD age. **Table S5.** Observed (expected) counts of *O. sativa japonica* retention mechanisms by SSD age. **Table S6.** Observed (expected) counts of *S. bicolor* retention mechanisms by SSD age. **Figure S1.** Monocot phylogeny used to infer SSD events. **Figure S2.** Distributions of Euclidean distances between gene expression profiles in *B. distachyon* (left), *O. sativa japonica* (middle), and *S. bicolor* (right). **Tables S4**–**6.** Contain observed (expected) counts of retention mechanism by SSD age in the three grass species, **Figure S1.** Contains the full monocot phylogeny used to infer SSD events, and **Figure S2.** Contains distributions of Euclidean distances between gene expression profiles in the three grass species. (PDF 2523 kb)

## Abbreviations

MYA: Million years ago
SSD: Small-scale duplication
TPM: Transcripts per million
WGD: Whole-genome duplication
