# Supplementary material for: Rapid functional divergence after small-scale gene duplication in grasses
Source: BMC Evol Biol. 2019 May 2;19:97. doi: 10.1186/s12862-019-1415-2 (PMC6498639; doi:10.1186/s12862-019-1415-2)
Supplement: Supplementary file 2 — Table S4. Observed (expected) counts of B. distachyon retention mechanisms by SSD age. Table S5. Observed (expected) counts of O. sativa japonica retention mechanisms by SSD age. Table S6. Observed (expected) counts of S. bicolor retention mechanisms by SSD age. Figure S1. Monocot phylogeny used to infer SSD events. Figure S2. Distributions of Euclidean distances between gene expression profiles in B. distachyon (left), O. sativa japonica (middle), and S. bicolor (right). Tables S4–6. Contain observed (expected) counts of retention mechanism by SSD age in the three grass species, Figure S1. Contains the full monocot phylogeny used to infer SSD events, and Figure S2. Contains distributions of Euclidean distances between gene expression profiles in the three grass species. (PDF 2523 kb) [file 12862_2019_1415_MOESM2_ESM.pdf]

Table S4. Observed (expected) counts of *B. distachyon* retention mechanisms by SSD age

	Conservation	Neofunctionalization (child)	Neofunctionalization (parent)	Specialization	P
H. vulgare	113 (115.05)	31 (28.93)	11 (11.44)	28 (27.58)	0.98
O. sativa japonica	36 (37.09)	8 (9.33)	5 (3.69)	10 (8.89)	0.85
S. bicolor	22 (18.86)	4 (4.74)	1 (1.88)	3 (4.52)	0.69

Duplication ages are given as divergence times from Figure 1 and are listed from youngest to oldest.

Table S5. Observed (expected) counts of *O. sativa japonica* retention mechanisms by SSD age

	Conservation	Neofunctionalization (child)	Neofunctionalization (parent)	Specialization	P
O. sativa indica	32 (31.82)	13 (11.28)	4 (4.83)	9 (10.07)	0.92
B. distachyon	108 (110.27)	39 (39.08)	15 (16.75)	39(34.90)	0.87
S. bicolor	18 (15.91)	4 (5.64)	5 (2.42)	2 (5.03)	0.14

Duplication ages are given as divergence times from Figure 1 and are listed from youngest to oldest.

Table S6. Observed (expected) counts of *S. bicolor* retention mechanisms by SSD age

	Conservation	Neofunctionalization (child)	Neofunctionalization (parent)	Specialization	P
Z. mays	103 (101.26)	27 (30.65)	5 (3.72)	22 (21.37)	0.82
S. italica	75 (72.24)	28 (21.87)	2 (2.65)	7 (15.24)	0.09
B. distachyon/ O. sativa japonica	40 (44.50)	11 (13.48)	1 (1.63)	17 (9.39)	0.07

Duplication ages are given as divergence times from Figure 1 and are listed from youngest to oldest.

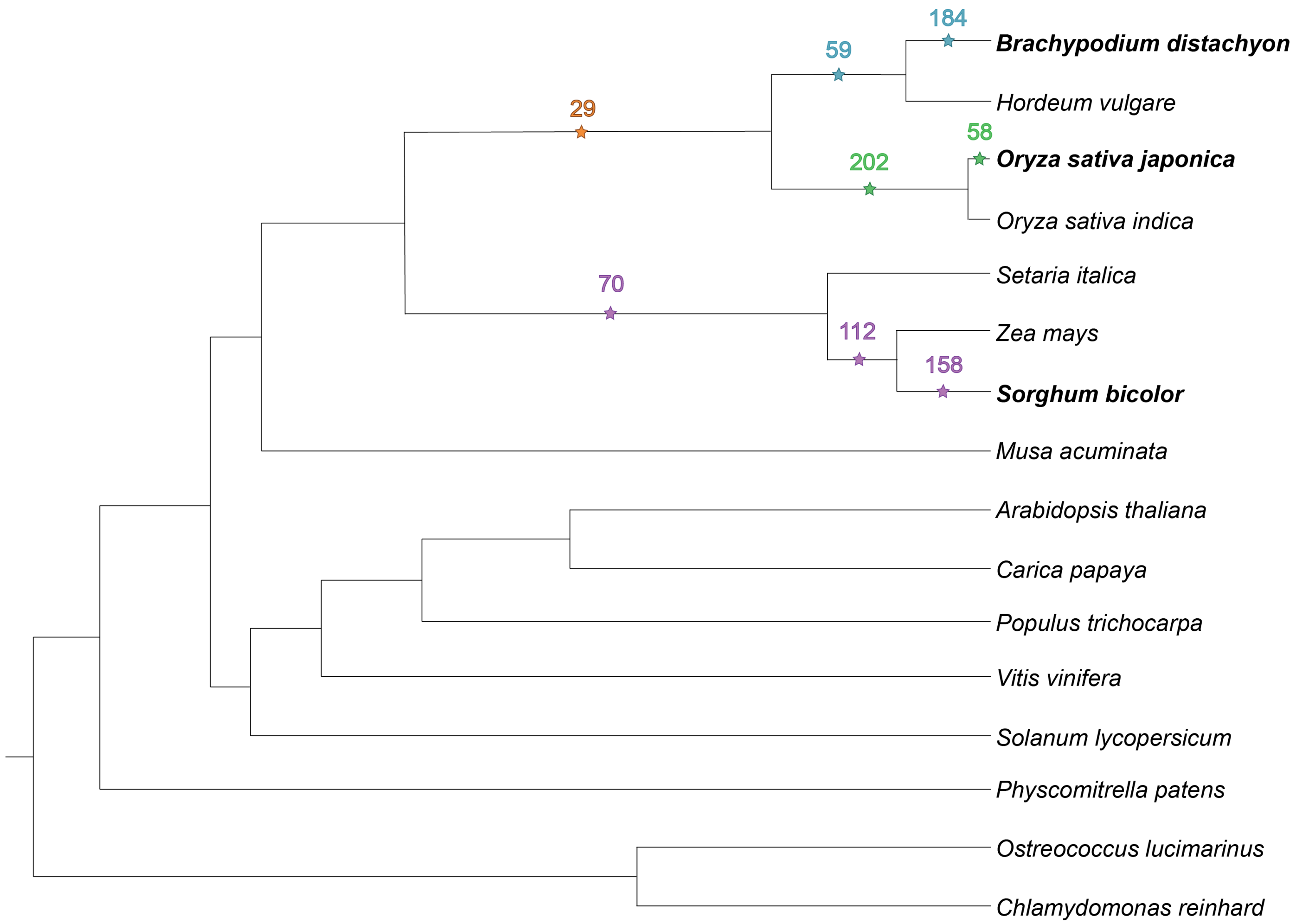

Figure S1. Monocot phylogeny used to infer SSD events. Numbers of duplicate gene pairs that arose via SSD along the *B. distachyon* (blue and orange stars), *O. sativa japonica* (green and orange stars), and *S. bicolor* (purple stars) lineages at specified divergence times on the monocot phylogeny.

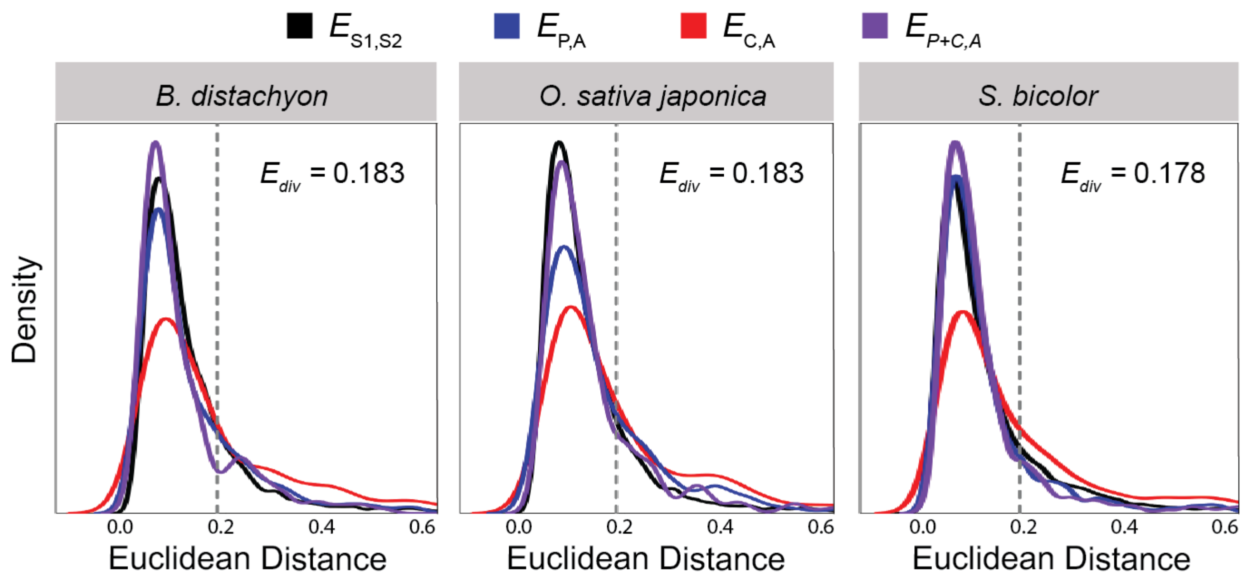

Figure S2. Distributions of Euclidean distances between gene expression profiles in *B. distachyon* (left), *O. sativa japonica* (middle), and *S. bicolor* (right). Distances were calculated between expression profiles of single-copy genes ($E_{\mathrm{S1,S2}}$, black), parent duplicates and ancestral genes ($E_{\mathrm{P,A}}$, blue), child duplicates and ancestral genes ($E_{\mathrm{C,A}}$, red), and parent and child duplicates combined and ancestral genes ($E_{\mathrm{P+C,A}}$, purple). Vertical dashed lines represent cutoffs (E_{div}) used to assess expression divergence of duplicate genes in each species.
